# Longitudinal reference ranges for maternal plasma laeverin, and its role as a potential biomarker of preeclampsia

**DOI:** 10.1186/s12884-016-1156-9

**Published:** 2016-11-25

**Authors:** Mona Nystad, Vasilis Sitras, Kari Flo, Christian Widnes, Åse Vårtun, Tom Wilsgaard, Ganesh Acharya

**Affiliations:** 1 Women’s Health and Perinatology Research Group, Department of Clinical Medicine, Faculty of Health Sciences, The Arctic University of Norway, Tromsø, Norway and Department of Obstetrics and Gynecology, University Hospital of North Norway, Tromsø, Norway; 2 Institute of Clinical Medicine, University of Oslo, and Department of Obstetrics, Fetal Medicine Unit, Akershus and Oslo University Hospital, Oslo, Norway; 3 Department of Community Medicine, Faculty of Health Sciences, The Arctic University of Norway, Tromsø, Norway; 4 Department of Clinical Sciences, Intervention and Technology, Karolinska Institute, Stockholm, Sweden

**Keywords:** Aminopeptidase-Q, ELISA, Laeverin, Plasma, Preeclampsia

## Abstract

**Background:**

Laeverin is a placenta-specific membrane-bound aminopeptidase. In this study we wanted to: 1) serially measure plasma levels of laeverin in healthy women during the second half of pregnancy and postpartum, 2) determine whether laeverin is differently expressed at 22–24 weeks in women who later develop preeclampsia compared to controls, 3) compare laeverin protein expression in placenta and umbilical vein serum in healthy and preeclamptic pregnancies at birth.

**Methods:**

Plasma was obtained serially, approximately every 4-weeks, from 53 healthy women with uncomplicated pregnancies during 22^+0^ to 39^+6^ weeks of gestation, and at 22–24 weeks from 15 women who later developed preeclampsia. Enzyme-linked immunosorbent assay was used to measure laeverin protein concentration. Serum from healthy non-pregnant premenopausal women (*n* = 10), menopausal women (*n* = 10) and men (*n* = 11) were used as negative controls. Protein extracts from placental tissue were obtained after birth from healthy- (*n* = 11) and preeclamptic women (*n* = 13). Paired umbilical artery and vein serum samples from the neonates (*n* = 10) of healthy mothers were also analyzed. Multilevel modeling was used to determine the reference centiles. Differences between groups were analyzed using Student’s *t*-test.

**Results:**

Healthy pregnant women at term (37–40 weeks) had significantly higher plasma levels of laeverin (mean 4.95 ± 0.32 ng/mL; *p* < 0.0001) compared to men (mean 0.18 ± 0.31 ng/mL), non-pregnant premenopausal women (mean 0.77 ± 0.26 ng/mL) and postmenopausal women (mean 0.57 ± 0.40 ng/mL). Maternal plasma laeverin levels decreased with advancing gestation, from 6.96 ± 0.32 ng/mL at 22–24 weeks to 4.95 ± 0.32 ng/mL at term (*p* < 0.0001) in uncomplicated pregnancies. Half of the women who developed preeclampsia had plasma laeverin levels below the 5^th^ percentile at 22–24 weeks gestation. However, laeverin levels were 1.6 fold higher in preeclamptic compared to healthy placentas (*p* = 0.0071). Umbilical venous samples of healthy neonates (*n* = 38) had higher (*p* = 0.001) mean levels of laeverin (16.63 ± 0.73 ng/mL), compared to neonates of preeclamptic (*n* = 14) mothers (12.02 ± 1.00 ng/mL). Postpartum plasma levels of laeverin decreased in healthy and preeclamptic women with a half-life of 3 and 5 days, respectively.

**Conclusions:**

Maternal plasma levels of laeverin decrease with advancing gestation during the second half of normal pregnancy and lower levels measured at 22–24 weeks might be associated with the development of preeclampsia later in gestation.

## Background

Laeverin (aminopeptidase Q) is a protein that is expressed by human villous trophoblasts [1]. More specifically, we showed previously that laeverin is expressed in the plasma membrane of villous trophoblasts in normal placentas, but in preeclamptic placentas it is ectopically expressed in the cytoplasm and secreted within microvesicles in the fetal capillaries [2]. These observations indicate that laeverin might be an attractive biomarker of preeclampsia. However, laeverin levels in maternal and fetal plasma during normal pregnancy and/or preeclampsia have not been investigated.

We hypothesized that plasma laeverin levels vary with gestational age during the second half of pregnancy and laeverin is differentially expressed in normal and preeclamptic pregnancies.

In this study we aimed to 1) serially measure plasma levels of laeverin in healthy women during the second half of pregnancy and postpartum to establish longitudinal reference ranges, 2) determine whether laeverin plasma levels measured at 22–24 weeks are different in women who later develop preeclampsia compared to controls, and 3) compare laeverin protein expression in placenta and umbilical vein serum in healthy and preeclamptic pregnancies at birth.

## Methods

The study was approved by the Regional Committee for Medical and Health Research Ethics-North Norway (REK Nord ref. number 5.2005.1386 (approved 27.09.05) and 2010/2058–4 (approved 13.01.05)) and informed written consent was obtained from all the participants.

### Study population

Fifty-three healthy pregnant women were recruited to the longitudinal arm of the study [3]. Blood samples were obtained serially between 22 and 40 weeks of gestation at approximately 4-weekly intervals (a total of 243 samples). Furthermore, blood samples were obtained from 42 of these healthy mothers at 1–6 days postpartum. Umbilical cord blood samples were obtained from 38 neonates immediately after birth.

Among a total of 641 pregnant women recruited to the cross-sectional arm of the study [3], 29 (4.5%) developed preeclampsia. Preeclampsia was defined as blood pressure (BP) of at least 140 mmHg (systolic) and/or 90 mmHg (diastolic) with proteinuria ≥1+ on dipstick, measured on at least two occasions 6 h apart after the 20th week of gestation. Women with pre-existing chronic hypertension, renal disease, lupus erythematosus, diabetes, and gestational hypertension without proteinuria were excluded. We had plasma samples from 15 of these women obtained at 22–24 weeks of gestation that were analyzed for laeverin. Postpartum (2–6 days) samples were also available from 4 of these women. Umbilical cord samples from 14 neonates from preeclamptic mothers were obtained immediately after birth.

Serum samples from eleven healthy men, ten healthy non-pregnant premenopausal women, and ten menopausal women were used as negative controls. All participants of the study were white Europeans.

Paired umbilical arterial and venous serum samples from the neonates of 10 healthy women with uncomplicated pregnancies were used to evaluate the arterial-venous gradient of laeverin concentration.

### Collection of serum and plasma samples

Peripheral blood samples were collected in EDTA or serum tubes, incubated at room temperature for 30 min and centrifuged at 1200×*g* for 10 min. Upper serum or plasma phase was collected and immediately frozen at −70 °C before further analysis.

### Placental tissue protein samples

Placental samples from 11 healthy and 13 preeclamptic women were used for protein isolation and laeverin quantification. Placental tissue samples were obtained immediately after delivery as described previously, snap frozen and stored for further use [4]. Placental tissue was cut in pieces and homogenized using MagNA Lyser Green Beads on MagNA Lyser (Roche, USA). Proteins were isolated using T-PER (Pierce, USA) with Complete Mini EDTA-free protease inhibitor cocktail (Roche, USA). Total protein concentration was measured using DC Protein Assay kit (BioRad, USA) in a ThermoMax Microplate Reader (Molecular Devices, USA). Total protein from 11 healthy placentas and 13 preeclamptic placentas was isolated.

### Human Aminopeptidase Q levels (AP-Q ELISA Kit)

Serum, plasma and protein samples were adjusted to room temperature. Enzyme-linked immunosorbent assay (ELISA) analysis was performed according to the manufacturer’s specifications (EIAab Science co., Ltd, Wuhan, China). ELISA is a specific and sensitive method for detection of small amounts of protein in serum [5, 6]. Measurements were performed in duplicate by a single investigator (MN). Optical density was measured using a ThermoMax Microplate Reader (Molecular Devices, USA) at 450 nm. Concentrations were estimated relating it to a standard curve of laeverin protein provided by the manufacturer. A calibration curve was plotted using absorbance (y-axis) against the concentration (x-axis). The mean absorbance was calculated for each set of standards, controls and samples and then subtracted the mean zero STD from each. Unknown concentration was determined by the interpolation method.

For quantification studies of the total placental protein samples from healthy and preeclamptic women 50 μg total protein was added to each well. Laeverin concentration was then derived from standard curves.

Total protein, extracted from placental samples, was used as positive controls of laeverin expression for validation of the kit. The lowest standard concentration of the assay was 2 ng/mL and the minimum detectable amount of the kit was 0.312 ng/mL (according to the manufacturer). The kit has sensitivity to detect concentrations down to 0.156 ng/mL. Intra- and inter-assay coefficients of variation (CV) calculated from duplicate patient samples in the concentration range 0.312–20 ng/mL were <6%.

### Statistics

Analysis of the longitudinal data was performed using SAS v.9.4 (SAS Institute Inc. Cary, NC). Multilevel modeling was used to estimate the reference percentiles [7]. SPSS software (IMB SPSS Statistics 22, Armonk, NY, USA) was used for the analysis of cross-sectional data. Sensitivity, specificity, positive predictive value and negative predictive value were calculated to evaluate usefulness of laeverin in predicting preeclampsia setting a cut-off value at ≤5 ng/mL. Differences between groups were evaluated using Student’s *t*-test for parametric variables and Mann-Whitney *U* test for non-parametric variables. Linear mixed model ANOVA was used to evaluate the differences in laeverin concentrations between different gestational weeks antenatally and different days post-partum. A *P*-value <0.05 was considered statistically significant.

## Results

### Phenotype of the study population

The phenotype of the study population is presented in Table 1. The demographic and clinical characteristics of women included in the longitudinal arm of the study have been reported previously.[3] Briefly, all the 53 participants were healthy women, and none of them developed any significant pregnancy complications. Follow up data on the course of pregnancy and perinatal outcome were available for all the participants.

**Table 1:** Phenotype of the study population (healthy pregnant women participating in the longitudinal arm of the study and women participating in the cross-sectional arm of the study who developed preeclampsia). Continuous variables are presented as mean ± SE or median (range) and categorical variables as n (%) as appropriate. Doppler ultrasonography was performed at 22–24 weeks of gestation

	Longitudinal study	Women who developed preeclampsia	*P* value
	Healthy women (*n* = 53)	Preeclampsia (*n* = 15)	
Maternal age, median (range), years	28.7 (18–39)	27.5 (18–40)	0.368
Body mass index before delivery, (kg/m^2^)	25.11 ± 0.41	25.75 ± 1.08	0.504
Primipara, n (%)	36 (68)	8 (53.3)	0.304
Mean arterial pressure, mmHg	79.40 ± 3.12	87.55 ± 1.28	0.003
Uterine artery pulsatility index (mean of the left and right side)	0.80 ± 0.02	1.24 ± 0.04	0.000
Middle cerebral artery pulsatility index	1.62 ± 0.06	1.82 ± 0.05	0.070
Umbilical artery pulsatility index	0.80 ± 0.02	1.24 ± 0.04	0.000
Gestational age at delivery, median (range), weeks	40 (37–42)	39 (32–41)	0.849
Cesarean section, n (%)	3 (5.66)	2 (13.33)	0.322
Neonatal birth weight, g	3562 ± 65	3329 ± 163	0.122
Placental weight, g	591 ± 17	584 ± 22	0.833
5 min APGAR score median (range)	10 (7–10)	9 (6–10)	0.472
Arterial cord blood pH	7.24 ± 0.02	7.23 ± 0.03	0.829
Arterial cord blood Base Excess, mmol/L	−5.65 ± 0.62	−3.59 ± 1.45	0.164
Venous cord blood pH	7.36 ± 0.01	7.35 ± 0.02	0.816
Venous cord blood Base Excess, mmol/L	−4.50 ± 0.45	−4.11 ± 1.35	0.731

Three (5.6%) women had cesarean section; two due to dystocia and one because of breech presentation. Six (11.2%) women had vacuum delivery; four due to failure to progress and two because of fetal distress in the second stage of labor.

Pregnant women participating in the cross-sectional arm of the study who developed preeclampsia (*n* = 15) had higher BMI, higher mean uterine artery pulsatility index, and were delivered at an earlier gestation (32 weeks (*n* = 1), 36 weeks (*n* = 1), 39 weeks (*n* = 3), 40 weeks (*n* = 3) 41 (*n* = 6) weeks and 42 (*n* = 1) weeks). Two (13%) women had cesarean section, three (20%) women were delivered by vacuum extraction and ten (67%) had normal vaginal delivery.

The baseline clinical characteristics and pregnancy outcome of the healthy (*n* = 11) and preeclamptic women (*n* = 13) whose placentas were used for placental laeverin quantification are presented in Table 2.

**Table 2:** Clinical characteristics and pregnancy outcomes of placentas used for tissue laeverin protein quantification. Continuous variables are presented as mean ± SE or median (range) and categorical variables as n (%) as appropriate. Doppler ultrasonography was performed < 24 h before delivery

	Healthy women (*n* = 11)	Preeclampsia (*n* = 13)	*P* value
Maternal age, median (range), years	27 (19–32)	29 (21–36)	0.562
Body mass index before delivery, (kg/m^2^)	23.49 ± 1.56	27.20 ± 1.66	0.118
Primipara, n (%)	7 (63.6)	6 (46.2)	0.532
Mean arterial pressure, mmHg	92.17 ± 1.91	130.85 ± 2.92	0.000
Uterine artery pulsatility index (mean of the left and right side)	0.66 ± 0.04	1.76 ± 0.34	0.028
Middle cerebral artery pulsatility index	1.06 ± 0.10	1.46 ± 0.12	0.006
Umbilical artery pulsatility index	0.73 ± 0.05	1.05 ± 0.08	0.028
Gestational age at delivery, median (range), weeks	40 (34–42)	35 (29–39)	0.024
Cesarean section, n (%)	1 (9.1)	9 (69)	0.005
Neonatal birth weight, g	3516 ± 201	2690 ± 248	0.018
Placental weight, g	622 ± 65	522 ± 54	0.247
5 min APGAR score median (range)	9 (8–10)	9 (5–10)	0.435
Arterial cord blood pH	7.28 ± 0.02	7.24 ± 0.03	0.457
Arterial cord blood Base Excess, mmol/L	−3.46 ± 1.52	−2.20 ± 1.52	0.582
Venous cord blood pH	7.35 ± 0.01	7.29 ± 0.02	0.043
Venous cord blood Base Excess, mmol/L	−4.16 ± 1.07	−1.72 ± 1.44	0.270

The neonates (*n* = 10) of healthy mothers whose umbilical cord blood samples were used to determine arterio-venous gradient of laeverin concentration were all delivered at term and had a mean birth weight of 3317 ± 200 g, placental weight 566 ± 38 g and normal umbilical artery pH (7.26 ± 0.04) and base excess (−1.05 ± 1.25 mmol/l).

### Serum laeverin levels in healthy men and non-pregnant controls

Healthy men (mean 0.18 ± 0.31 ng/mL), non-pregnant premenopausal women (mean 0.77 ± 0.26 ng/mL) and postmenopausal women (mean 0.57 ± 0.40 ng/mL) had very low levels of laeverin (mean 0.50 ± 0.19 ng/mL).

### Plasma laeverin levels in healthy women during the second half of pregnancy

**Fig. 1:**
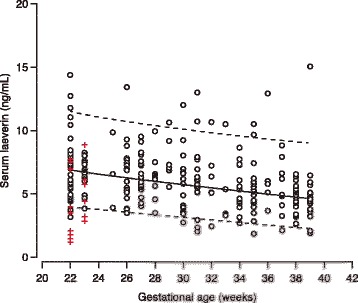
Maternal plasma laeverin concentration (ng/ml) in healthy pregnant women during the second half of pregnancy and maternal plasma laeverin concentration at 22–24 weeks in women who later developed preeclampsia. The solid line represents the 50th percentile and the interrupted lines represent the 5th and 95th percentiles for the gestational age. Circles represent healthy pregnant women and red crosses laeverin protein expression at 22–24 weeks in women who later developed preeclampsia

**Fig. 2:**
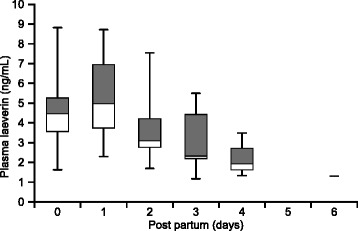
Box-and-whisker plots showing plasma laeverin levels (ng/ml) levels in 41 healthy women at gestational week 37–39 (timepoint 0) and at 1–4 days post-partum. Grey boxes represent upper quartile, white boxes represent lower quartile and the line between them represents the median value of each series. Whiskers represent the highest and lowest points within the range

Gestational age specific longitudinal references ranges for maternal plasma laeverin concentration during the second half of pregnancy are presented in Table 3 and Fig. 1. Laeverin levels decreased with advancing gestation, from 6.96 ± 0.32 ng/mL at 22–24 weeks to 4.95 ± 0.32 ng/mL at term (37–40 week) (*p* < 0.0001). We found significantly lower laeverin levels on postpartum day 2 (*p* = 0.0454) and 3 (*p* = 0.01) compared to the antepartum laeverin levels, but the difference was not statistically significant on the first postpartum day. Laeverin levels decreased further after delivery from a median value of 4.98 ng/mL on 1st day postpartum to 1.31 ng/mL on 6th day postpartum (Fig. 2). We performed mixed model ANOVA and found a significant difference (*p* = 0.0055) in laeverin levels between different postpartum days. In the postpartum period, in healthy women, laeverin protein half-life (t_1/2_) was 3 days.

**Table 3:** Longitudinal reference ranges for maternal plasma laeverin concentration during the second half of pregnancy

Gestation (weeks)	Percentiles						
	2.5^th^	5^th^	10^th^	50^th^	90^th^	95^th^	97.5^th^
22	3.6	4.0	4.5	6.9	10.3	11.5	12.6
23	3.5	3.9	4.4	6.7	10.1	11.3	12.4
25	3.3	3.7	4.2	6.4	9.7	10.9	12.0
26	3.2	3.6	4.1	6.3	9.5	10.7	11.8
27	3.1	3.5	3.9	6.1	9.4	10.5	11.6
28	3.0	3.4	3.8	6.0	9.2	10.4	11.5
29	2.9	3.3	3.7	5.9	9.1	10.2	11.3
30	2.8	3.1	3.6	5.7	8.9	10.1	11.2
31	2.7	3.0	3.5	5.6	8.8	9.9	11.0
32	2.6	2.9	3.4	5.5	8.6	9.8	10.9
33	2.5	2.8	3.3	5.3	8.5	9.7	10.8
34	2.4	2.7	3.2	5.2	8.4	9.6	10.7
35	2.3	2.6	3.1	5.1	8.3	9.4	10.6
36	2.2	2.5	3.0	5.0	8.2	9.3	10.5
37	2.1	2.5	2.9	4.9	8.0	9.2	10.4
38	2.1	2.4	2.8	4.8	7.9	9.1	10.3
39	2.0	2.3	2.7	4.6	7.8	9.0	10.2

### Plasma laeverin in women developing preeclampsia

Plasma laeverin concentrations of 15 women at 22–24 weeks of gestation, who later developed preeclampsia, plotted against the normal longitudinal reference curves, are shown in Fig. 1 (median concentration of 3.99 +/−2.51 (SD) and *p* = 0.006). In four women (27%), plasma laeverin levels were between 50-75^th^ percentile, three women (20%) had laeverin levels below the 50^th^ percentile and eight (53%) had below the 5^th^ percentile. In women developing preeclampsia, the plasma laeverin concentration decreased postpartum from 4.27 ng/mL on 2^nd^ day to undetectable level on the 6^th^ day after delivery. The half-life of postpartum laeverin was 5 days in preeclamptic women. Among the 15 preeclamptic women, only one had severe (i.e., BP ≥ 160/110 with +2 proteinuria) early-onset (i.e., less than 34 weeks gestation) disease. The serum laeverin levels of this woman were not significantly different compared to the rest of the preeclamptic women (*p* = 0.069).

With a cut-off value for laeverin concentration set at ≤5 ng/mL, the sensitivity, specificity, positive predictive value and negative predictive value for the prediction of preeclampsia were 0.60, 0.79, 0.82 and 0.88, respectively.

### Placental tissue laeverin protein quantitation

Placental tissue of 83 mg gave 5 μg total protein, of which 0.26 ng was laeverin protein. Laeverin protein concentration was 1.6 fold higher in preeclamptic placentas (mean 8.29 ± 0.73 ng/mL) compared to healthy placentas (mean 5.18 ± 0.75 ng/mL) (*p* = 0.0071).

### Umbilical cord blood laeverin concentration

The neonates of the healthy mothers had a mean laeverin concentration of 16.63 ± 0.73 ng/mL in the umbilical vein serum samples with no significant difference between girls (*n* = 22) and boys (*n* = 16) (16.20 ± 1.05 ng/mL versus 17.22 ± 0.99 ng/mL, respectively; *p* = 0.111). In the neonates of women who had preeclampsia (*n* = 14), the mean laeverin level in the umbilical vein was significantly lower (12.02 ± 1.00 ng/mL) compared to healthy pregnancies (*p* = 0.001).

### Arterial-venous gradient of laeverin in the umbilical cord blood

Concentration of laeverin was checked in paired umbilical artery and vein serum samples of 10 healthy neonates of mothers who had uncomplicated pregnancies. The difference between umbilical artery (mean, 14.12 ± 0.91 ng/mL) and vein (mean, 15.46 ± 1.17 ng/mL) was not statistically significant.

## Discussion

This is the first study to investigate gestational age-associated longitudinal changes in plasma laeverin levels in healthy women with uncomplicated pregnancies. We found that plasma laeverin levels gradually decrease with advancing gestation during the second half of pregnancy and are undetectable few days after delivery. The reasons for decreasing levels of laeverin are unclear. As laeverin is a placenta-specific protein, it is likely to have an important role in the process of placentation during early pregnancy, but less might be required after this process is complete. Indeed, we showed previously that laeverin effects migration and invasion of first trimester trophoblast cells [2]. Alternatively, as total plasma volume increases normally during pregnancy, the reduction of plasma laeverin levels might be seen due to dilution effect.

Furthermore, we found lower plasma levels of laeverin at 22–24 weeks in women who later developed preeclampsia compared to healthy controls. These women most likely had shallow placentation. We have previously shown that laeverin is overexpressed in preeclamptic placenta both at mRNA as well as protein level [2, 4]. This leads us to speculate that laeverin might be trapped in the placenta during the development of preeclampsia. In fact, tissue protein quantification showed a 1.6 fold increase of laeverin protein in preeclamptic compared to healthy placentas. This is despite of generally reduced placental size in preeclamptic pregnancies [8]. We have previously shown an abnormal expression of laeverin in cytoplasm of trophoblast cells in preeclamptic placentas, which may indicate deregulation or improper folding of the protein [2]. It is known that preeclampsia leads to increased apoptosis of cytotrophoblasts or cell death [9, 10], which might explain why laeverin accumulates in preeclamptic placentas. Furthermore Horie et al. demonstrated that laeverin is intensely expressed on the surface of extravillous trophoblasts invading maternal decidua and vessels [11]. Since defective endovascular invasion of extravillous trophoblast has been considered to be a primary cause of preeclampsia it is possible that reduced number of extravillous trophoblast colonizing inside the maternal vessels could be one of the causes of lower plasma laeverin at 22–24 weeks of gestation in the women who later developed preeclampsia.

Preeclampsia is associated with increased apoptosis or cell death of cytotrophoblasts. The exact molecular mechanisms behind these phenomena are not known. It is suggested that endoplasmatic reticulum stress can lead to cell apoptosis and protein degradation [12, 13]. Therefore, we hypothesize that the observed accumulation of laeverin in the cytoplasm of cytotrophoblasts of preeclamptic placentas could be explained by defective protein glycosylation and/or misfolding occurring in the endoplasmatic reticulum [13, 14].

Another possible explanation of the reduced laeverin protein expression in the maternal circulation might be that laeverin is trapped in microvesicles in the fetal capillaries in the placenta of preeclamptic patients [2]. However, even if these microvesicles were transported to the maternal circulation, we would not be able to detect laeverin inside them, since our present ELISA-method did not include isolation and disruption of microvesicles. Moreover, we found similar laeverin concentrations in the umbilical artery and vein indicating that there is no significant production or metabolism of laeverin in the fetus. This is in line with the previous studies, demonstrating that placenta is the major organ producing laeverin [1].

Our study has some limitations. Samples were stored for up to 5 years at −70°C and some samples were thawed once. However minimal differences in laeverin levels were found when thawed and non-thawed samples were compared (data not shown). In a study by Rana et al. minimal effect on plasma and serum quality was seen when samples were thawed several times [15]. Further, we were able to include only a small number of samples from preeclamptic women. Therefore, this study should be reproduced in a bigger cohort, and preferably first trimester samples should also be included.

We used a commercial kit to measure laeverin concentration, which has not been previously validated for use in the context of pregnancies and placenta samples. Therefore, we performed extensive validation prior to analysis. Healthy men, non-pregnant premenopausal women and menopausal women had a very low expression of laeverin indicating that laeverin is a placenta-specific protein and the aminopeptidase Q ELISA kit appeared to be a versatile test for the quantitation of laeverin in serum-, plasma- and placental tissue protein samples.

## Conclusions

In conclusion, our longitudinal study showed that the maternal plasma laeverin concentration gradually decreases during the second half of normal pregnancy. Women who later developed preeclampsia had lower levels of laeverin compared to healthy controls at 22–24 weeks of gestation in our cohort. Further research is needed to investigate the potential role of laeverin as a predictive biomarker of preeclampsia.
